# Supplementary material for: Cross-cultural adaptation and content validation of the Infant Feeding Intentions Scale for Thai pregnant women
Source: PLOS Glob Public Health. 2023 Mar 29;3(3):e0000729. doi: 10.1371/journal.pgph.0000729 (PMC10058147; doi:10.1371/journal.pgph.0000729)
Supplement: S1 Text — (PDF) [file pgph.0000729.s001.pdf]

S1 Text

Demographic questionnaire and T-IFI scale

ข้อมูลส่วนบุคคล

คำชี้แจง โปรดทำเครื่องหมาย (✓) ลงในช่องว่างหน้าข้อความ หรือเติมข้อความ ที่ท่านเห็นว่าเหมาะสมและตรงกับความเป็นจริงมากที่สุด

1. อายุ................................. ปีเต็ม

2. สถานภาพทางครอบครัว

( ) โสด ( ) คู่ ( ) หม้าย/หย่า/แยก

3. ระดับการศึกษา

( ) ประถมศึกษา ( ) มัธยมศึกษา

( ) อาชีวศึกษา/อนุปริญญา ( ) ปริญญาตรี

( ) สูงกว่าระดับปริญญาตรี ( ) อื่น ๆ ระบุ.............................

4. รายได้ครอบครัวเฉลี่ยต่อเดือน ....บาท

5. อาชีพ

( ) ข้าราชการ/ พนักงานรัฐวิสาหกิจ ( ) ค้าขาย/ธุรกิจส่วนตัว/เจ้าของกิจการ

( ) พนักงานบริษัทเอกชน ( ) เกษตรกรรม/ประมง

( ) รับจ้าง ( ) อาชีพอิสระ

( ) นักเรียน/นักศึกษา ( ) ไม่ประกอบอาชีพ

แบบสอบถามความตั้งใจในการให้นมลูก

โปรดทำเครื่องหมาย (✓) ในช่อง ❑ ที่ตรงกับความคิดเห็นของคุณมากที่สุด

คุณอาจจะยังไม่ทราบอย่างแน่ชัดถึงแผนการให้นมลูกของคุณ แต่คุณอาจจะมีแนวคิดว่า คุณจะให้นมลูกด้วยวิธีใด ฉันจะอ่านข้อความเกี่ยวกับการให้นมลูกของคุณ เพื่อให้คุณเลือกคำตอบที่ตรงกับความคิดเห็นของคุณมากที่สุด เกี่ยวกับแผนการให้นมลูกของคุณในปัจจุบัน และความเป็นไปได้ที่คุณจะทำตามแผน

ความตั้งใจในการให้นมลูก	เห็นด้วยอย่างยิ่ง	เห็นด้วยบางส่วน	ไม่แน่ใจ	ไม่เห็นด้วยบางส่วน	ไม่เห็นด้วยอย่างยิ่ง
1. ฉันวางแผนว่า จะให้นมผสมกับลูกอย่างเดียว โดยไม่เลี้ยงลูกด้วยนมแม่เลย	❑	❑	❑	❑	❑
2. ฉันวางแผนว่า จะเลี้ยงลูกด้วยนมแม่ หรืออย่างน้อยพยายามลองเลี้ยงลูกด้วยนมแม่	❑	❑	❑	❑	❑
3. ตั้งแต่แรกเกิดจนถึง 1 เดือนแรก ฉันจะเลี้ยงลูกด้วยนมแม่ โดยไม่ให้นมผสม หรือนมชนิดอื่นเลย	❑	❑	❑	❑	❑
4. ตั้งแต่แรกเกิดจนถึง 3 เดือนแรก ฉันจะเลี้ยงลูกด้วยนมแม่ โดยไม่ให้นมผสม หรือนมชนิดอื่นเลย	❑	❑	❑	❑	❑
5. ตั้งแต่แรกเกิดจนถึง 6 เดือนแรก ฉันจะเลี้ยงลูกด้วยนมแม่ โดยไม่ให้นมผสม หรือนมชนิดอื่นเลย	❑	❑	❑	❑	❑

The Thai Infant Feeding Intentions (T-IFI) Scale © 2022 by Ratchanok Phonyiam, Donruedee Kamkhoad, and Aunchalee E.L. Palmquist is licensed under CC BY 4.0. To view a copy of this license, visit http://creativecommons.org/licenses/by/4.0/
