# Supplementary material for: Cross-cultural adaptation and content validation of the Infant Feeding Intentions Scale for Thai pregnant women
Source: PLOS Glob Public Health. 2023 Mar 29;3(3):e0000729. doi: 10.1371/journal.pgph.0000729 (PMC10058147; doi:10.1371/journal.pgph.0000729)
Supplement: Attachment — Submitted filename: PGPH-D-22-00762 Response to Reviewers.docx [file pgph.0000729.s004.docx]

**PGPH-D-22-00762
Cross-Cultural Adaptation and Content Validation of the Infant Feeding Intentions Scale
for Thai Pregnant Women**

Dear Editor,

We appreciate the opportunity to address the comments and to revise this manuscript. Below please find item-by-item responses to those comments, which are included verbatim. We believe the manuscript has been considerably strengthened with these revisions.

**Responses to Journal Requirements:**

1. Please include a complete copy of PLOS’ questionnaire on inclusivity in global research in your revised manuscript. Our policy for research in this area aims to improve transparency in the reporting of research performed outside of researchers’ own country or community. The policy applies to researchers who have travelled to a different country to conduct research, research with Indigenous populations or their lands, and research on cultural artefacts. The questionnaire can also be requested at the journal’s discretion for any other submissions, even if these conditions are not met.  Please find more information on the policy and a link to download a blank copy of the questionnaire here: https://journals.plos.org/globalpublihealth/s/best-practices-in-research-reporting. Please upload a completed version of your questionnaire as Supporting Information when you resubmit your manuscript.
**⬝ Response:** We completed a questionnaire as supporting information and included “inclusively in global research” as suggested.

2. Please amend your detailed online Financial Disclosure statement. This is published with the article. It must therefore be completed in full sentences and contain the exact wording you wish to be published.
a. State the initials, alongside each funding source, of each author to receive each grant.
b. State what role the funders took in the study. If the funders had no role in your study, please state: “The funders had no role in study design, data collection and analysis, decision to publish, or preparation of the manuscript.”
**⬝ Response:** Please see a revision below:

**Funding statement**
 RP received the Alpha Alpha Chapter of Sigma Theta Tau International Research Grant to complete this work. The funders had no role in study design, data collection and analysis, decision to publish, or preparation of the manuscript.

3. Please update your online Competing Interests statement. If you have no competing interests to declare, please state: “The authors have declared that no competing interests exist.”
**⬝ Response:** We made changes as suggested.

4. In the online submission form, you indicated that “The datasets available from the corresponding author on reasonable request.”. All PLOS journals now require all data underlying the findings described in their manuscript to be freely available to other researchers, either 1. In a public repository, 2. Within the manuscript itself, or 3. Uploaded as supplementary information.
This policy applies to all data except where public deposition would breach compliance with the protocol approved by your research ethics board. If your data cannot be made publicly available for ethical or legal reasons (e.g., public availability would compromise patient privacy), please explain your reasons by return email and your exemption request will be escalated to the editor for approval. Your exemption request will be handled independently and will not hold up the peer review process, but will need to be resolved should your manuscript be accepted for publication. One of the Editorial team will then be in touch if there are any issues.
**⬝ Response:** We added a supplement file (see S1 text). This supplement information included the participants demographic and T-IFI dataset.

5. Please include a title page at the beginning of your manuscript file, that lists full author names and institute addresses. Do not upload as a separate file.  
**⬝ Response:** We included a title page at the beginning of our revised manuscript as suggested.

6. Please provide separate figure files in .tif or .eps format and remove any figures embedded in your manuscript file. Please also ensure that all files are under our size limit of 10MB.
For more information about how to convert your figure files please see our guidelines: https://journals.plos.org/globalpublichealth/s/figures
**⬝ Response:** We converted Figure 1 in .tif format.

**Responses to Reviewer 1:**
Comment 1. The authors described in the title and objectives of the article that the "validation" of the instrument will be carried out, however the authors only perform the pre-test stage with 30 pregnant women, which characterizes only an initial stage of psychometric analysis or for some authors as the end of cultural adaptation. Therefore, I recommend that the authors change the title and objective of the work, indicating that the cultural adaptation of the instrument was carried out.
**⬝ Response:** Thank you very much for your suggestion. We made changes on our manuscript title as “Cross-Cultural Adaptation and Content Validation of the Infant Feeding Intentions Scale for Thai Pregnant Women”

Comment 2. Regarding the calculation of the Content Validity Index, I recommend that the authors indicate the total reference value adopted for the index.
**⬝ Response:** We added a statement describing the content validity index (CVI) calculation on page 7.

Item Content validity Index (I-CVI) was calculated by the number of experts who rated this specific item as content valid (3 or 4 scores), divided by the total number of the experts. Scale Content Validity Index (S-CVI) was calculated by the number of items that were rated as content valid (3 or 4 scores) by the experts, divided by the total numbers of items [20].

Comment 3. What is the reference adopted for the selection of 30 pregnant women in the pre-test stage? I recommend indicating the reference of Beaton et al., 2000, which indicates the inclusion of 30 subjects in this stage.
**⬝ Response:** We added a citation [19]. Please see the revised submission in the attached document.

Comment 4. In the first paragraph of the results, the authors describe the sample number of the pre-test stage, however, to follow a logical sequence, I recommend that you start the Results topic with the description of the cultural adaptation stage.
**⬝ Response:** Thank you so much for your detailed comments. We revised the first paragraph of the results. Please see the revised submission in the attached document.


Comment 5. At the conclusion of the study, the authors state "Satisfactory internal consistency reliability and content validity of the scale were demonstrated in this study. Consequently, our T-IFI scale can be applicable in research and clinical settings for Thai-speaking populations", however the scale has not yet been fully validated, so the authors should not make such a claim.
**⬝ Response:** Thank you for pointing this out. We agreed with your comments and made changes on the conclusion on page 12.

**Responses to Reviewer 2:**
Reviewer #2: This manuscript describes a validation study conducted to validate a translated version of the Infant Feeding Intentions Scale adapted to the Thai context. The paper follows a rigorous methodology and is clear and concise in its presentation. Figure 1 is especially well done. The approach is an excellent template for other translations of the IFI for use in other contexts.
Limitations of the study are clearly articulated, which include limited demographic generalizability and lack of follow up data to enable validation of predictive value of Thai IFI. Thus, overall, this reviewer has only minor comments.
**⬝ Response:** Thank you very much.

1. Table 1--consider a footnote to explain the calculation of I-CVI and S-CVI indexes. These are described in the text but is more informative when juxtaposed against the results summarized in Table 1.
**⬝ Response:** I added a footnote according to your suggestion.

2. In the results, consider first describing the participant characteristics and then presenting the ICR results.
**⬝ Response:** We made changes as suggested.

3. The word 'Breastfeeding' with no qualifier (i.e., without 'exclusive' in front) is used throughout the manuscript when the original scale is designed to assess strength of EXCLUSIVE breastfeeding intention. Please clarify if the Thai adapted scale is designed to quantify any breastfeeding intention or exclusive breastfeeding intention? Was it an oversight or intentional to use 'breastfeeding' and not 'exclusive breastfeeding?'
**⬝ Response:** Our T-IFI scale is designed to measure exclusive breastfeeding intention. We added a statement to deepen our response on page 5.

4. What is the rationale for not including the translated scale as part of the manuscript?
**⬝ Response:** We uploaded our T-IFI after the first submission because our team was preparing for the license under CC BY 4.0. We have our T-IFI attached to this revision.

**Responses to Additional Journal Requirements:**

1. Thank you for your recent responses to requests for additional information. Our internal checks have shown that the following has not yet been addressed - unfortunately, we cannot proceed with your manuscript until you complete this request. Thank you for your understanding:
Please include a complete copy of PLOS’ questionnaire on inclusivity in global research in your revised manuscript. Our policy for research in this area aims to improve transparency in the reporting of research performed outside of researchers’ own country or community. The policy applies to researchers who have travelled to a different country to conduct research, research with Indigenous populations or their lands, and research on cultural artefacts. The questionnaire can also be requested at the journal’s discretion for any other submissions, even if these conditions are not met.  Please find more information on the policy and a link to download a blank copy of the questionnaire here: <https://journals.plos.org/globalpublihealth/s/best-practices-in-research-reporting>. Please upload a completed version of your questionnaire as Supporting Information when you resubmit your manuscript.
**⬝ Response:** We completed the PLOS’ questionnaire and uploaded it as supporting information.

2. Please include a separate legend for Figure 1 in your manuscript.
**⬝ Response:** Thank you for your suggestion. We added the explanation of the Figure 1.

3. Please remove your figure from within your manuscript file, leaving only the individual TIFF image file. These will be automatically included in the reviewer’s PDF.
**⬝ Response:** We removed Figure 1 from our manuscript.

4. We have noticed that you have uploaded Supporting Information files, but you have not included a list of legends. Please add a full list of legends for your Supporting Information files after the references list.

**⬝ Response:** We added a list of legends for supporting information files. See a revision below:

**Supporting information**

S1 Text. Demographic questionnaire and T-IFI scale.

S2 Text. Checklist.

S3 Text. Dataset.

We appreciate your thoughtful comments and suggestions. We believe through the revision process; the manuscript has been improved. Thank you in advance for your consideration.

I look forward to hearing from you.

Sincerely,

Authors
