# Supplementary material for: Cross-cultural adaptation and content validation of the Infant Feeding Intentions Scale for Thai pregnant women
Source: PLOS Glob Public Health. 2023 Mar 29;3(3):e0000729. doi: 10.1371/journal.pgph.0000729 (PMC10058147; doi:10.1371/journal.pgph.0000729)
Supplement: Attachment — Submitted filename: PGPH-D-22-00762R1 Response to Reviewers.docx [file pgph.0000729.s005.docx]

**PGPH-D-22-00762R1**

**Cross-Cultural Adaptation and Content Validation of the Infant Feeding Intentions Scale
for Thai Pregnant Women**

Dear Reviewers,

We appreciate the opportunity to address the comments and to revise this manuscript. Below please find item-by-item responses to those comments, which are included verbatim. We have indicated all revisions by using text changes, and all page and line numbers refer to locations in the revised manuscript (PGPH-D-22-00762R1). We believe the manuscript has been considerably strengthened with these revisions.

| **Editor Comment** | **Author Response** |
| --- | --- |
| Thank you for submitting your manuscript to PGPH for publication. It has been reviewed by two independent reviewers. Although one recommended that the paper should be accepted, the other suggested minor revision. Their concern focuses on the sample size (I don't think this is an issue, as the study is not intended to be generalizable) and its calculation. I will suggest you adequately address their comments to avoid a delay in the publication of your manuscript. Also, read through the entire manuscript carefully to address any grammar, diction and structural issues. | Thank you for your thoughtful comments and suggestions.  We have carefully considered all the issues brought to our attention and made necessary changes point by point. Please see the revised submission in the attached document. |

| **Reviewer 2 Comment** | **Author Response** |
| --- | --- |
| All reviewer concerns were addressed.  I recommend acceptance of this manuscript. | Thank you very much. |

| **Reviewer 3 Comment** | **Author Response** |
| --- | --- |
| The manuscript presents an important tool for testing the Infant Feeding Intentions for pregnant women towards breastfeeding for six months, which can be a useful tool for intention prediction. In the materials and methods; the study is methodological and cross-sectional where the first one consist of translation and cross-cultural adaptation of the IFI scale for Thai pregnant women and the later was a quantitative assessment of its psychometric properties. | Thank you very much. |
| The manuscript methodology is not well presented especially in the quantitative part.  1. How was the sample size calculated (i.e. 30 Women?), what were the criteria used for the calculation? | Thank you for your questions. The purpose of this study is to validate a translated and culturally adapted version of a scale. The sample size of 30 women was derived from the Beaton and colleagues (2000) guideline. We added a detailed statement and a citation. Please see Line 199-200. |
| 2. How were these 30 participants found? which platform were used to select them? Does this account for any biasness in the results for a certain socioeconomic group? | Thank you so much for pointing this out.  We described our recruitment process to deepen our response.  Yes, we randomly approached potential participants to minimize biases and to reach women from various socioeconomic backgrounds. Please see Line 200-210. |
| 3. The results part is also weak; there author indicated that they have used SPSS to test for normality and outliers; but in the results they have not presented this aspects. | Thank you for your comment.  Before analyzing our qualitative data, we assessed for normality and outliers on continuous measures. The results showed that maternal age had a normal distribution. Monthly household income had right-skewed distribution indicating most participants tended to have a lower income than average. The T-IFI scores had left-screwed distribution indicating most participants had higher infant feeding intention than average. Outliers were found from ones with higher income and those with lower T-IFI score.  We add the results from SPSS testing normality and outliers as suggested. Please see Line 318-320 and Line 408-409. |
| 4. Due to smaller sample size; the categories should be reduced and remove the ones with no observation (i.e. agriculture in the employment status. | Thank you so much for pointing this out. We removed the results with no observation as suggested. Please see Table 2 Page 10. |
| 5. What do the authors draw from the IFI and socio-demographic characteristics? Is there any story behind? Can the IFI be attributed by other factors among Thai Pregnant Women? | Thank you very much. We appreciate your questions. We added our explanations in the limitations. Please see Line 427-429 and 443-446. |
| 6. The results fails to justify the adaptation part of the IFI. | We added our justification as suggested. Please see Line 288-289, 293-294, and 313-315. |

We appreciate your thoughtful comments and suggestions. We believe through the revision process; the manuscript has been improved. Thank you in advance for your consideration.

We look forward to hearing from you.

Sincerely,

Authors
