# Supplementary material for: Cross-cultural adaptation and content validation of the Infant Feeding Intentions Scale for Thai pregnant women
Source: PLOS Glob Public Health. 2023 Mar 29;3(3):e0000729. doi: 10.1371/journal.pgph.0000729 (PMC10058147; doi:10.1371/journal.pgph.0000729)
Supplement: Attachment — Submitted filename: PGPH-D-22-00762R2 Response to Reviewers.docx [file pgph.0000729.s006.docx]

**PGPH-D-22-00762R2**

**Cross-Cultural Adaptation and Content Validation of the Infant Feeding Intentions Scale
for Thai Pregnant Women**

Dear Editor and Reviewers,

We appreciate the opportunity to address the comments and to revise this manuscript. Below please find item-by-item responses to those comments, which are included verbatim. We have indicated all revisions by using track changes, and all page and line numbers refer to locations in the revised manuscript (PGPH-D-22-00762R2). We believe the manuscript has been considerably strengthened with these revisions.

| **Editor Comment** | **Author Response** |
| --- | --- |
| Thank you for submitting your work to PGPH. Although one reviewer recommended that the manuscript be accepted for publication, another felt there are some minor issues that need to be addressed to make it publishable. I suggest you carefully addressed the reviewer's comments to enable me take a final decision on your manuscript. Also proofread it to address possible grammar/diction issues. | Thank you for your thoughtful suggestions.  We have carefully considered all the issues brought to our attention and made necessary changes point by point. Please see the revised submission in the attached document. |

| **Reviewer 3 Comment** | **Author Response** |
| --- | --- |
| All comments were addressed. On the final tone, please ensure proper grammar i.e. the use of "screwed", I think it meant "skewed". | Thank you so much for pointing this out.  Please see a revision Line 288. |

| **Reviewer 4 Comment** | **Author Response** |
| --- | --- |
| Thank you for the invitation to review this manuscript. This study is a Thai translation and cultural adaptation of the Infant Feeding Intentions Scale. Translating evidence-based questionnaires for LMIC and non-English speaking populations is a really important task. Given the need for quantifying, assessing, and addressing low breastfeeding rates in upper middle income settings like Thailand, this work has real utility and generalizability to other contexts. It would be great to test this in the future with a larger sample of Thai women. In general, this study is methodologically sound and should eventually be accepted. | Thank you for your thoughtful comments.  We hope to test this T-IFI with Thai pregnant women in our future research. |
| ABSTRACT 1. Line 72-74, “Content Validity Index was equal to 1” and “The expert review committee validated a cultural adaptation”: Could this be a bit more descriptive? “Both Item and Scale Content Validity Indices equaled 1, scored by 5 experts, who also validated the content for cross-cultural adaptation.” | Please see a revision Line 72-73. |
| 2. Line 76, “culturally responsive” revise to “culturally appropriate” or “culturally adapted”? | Please see a revision Line 77. |
| INTRODUCTION 3. Line 100-101, “although several interventions…”: This paragraph does not explain the main factors why breastfeeding is low—what are the main factors? This may also give strength to the rationale/importance of this study—e.g., “the T-IFI is useful in addressing low breastfeeding rates by helping to understand breastfeeding intent”? | We added a statement describing factors that contributed low breastfeeding rate (Line 107-109) and a justification for why the T-IFI can be useful (Line 140-141). |
| 4. Line 106-108, “Prenatal breastfeeding education…”: perhaps this sentence is better to start the next paragraph. | Please see a revision Line 113. |
| 5. Lines 111-113, “Evidence from the US…”: What does this mean for Asian populations? Thailand or southeast Asia? | Please see a revision Line 116-117. |
| 6. Line 116 “hence further guides” revise to "may help guide" | Please see a revision Line 121. |
| MATERIALS AND METHODS 7. Lines 135-139, “Written authorization…”: Should this information be included at the end of the methods section? Should you include the official ethics IRB approval numbers? | We added the IRB approval numbers (Line 152-153). We decided to keep the information related to authorization from the scale developer and IRB approvals at the beginning of our methods section. |
| 8. Line 177 “PR”: RP or PR or is this an acronym for something else? If it is in reference to the corresponding author please make sure the correct initials in the rest of the document. | Thank you for pointing this out. We made changes. Please see Line 193 and 208**.** |
| 9. Line 181, revise “In the stages of content validation,” to “For content validation,.” | Please see a revision Line 197. |
| 10. Line 196 “could be necessary” revise to “"a sample of 30 participants is considered sufficient for testing a scale". It seems that the authors have been asked this question about sample size from other reviewers. This answer is acceptable as the authors used a framework and mention in the Discussion that there is a need to test with a larger sample. The authors may also consider adding to the discussion how the sample size in this study compares to the original sample size in testing/validity of the IFI and how this might compare to other sample sizes for translations/cross-cultural adaptations of this IFI scale in other populations. | Please see a revision Line 214.  We also added the information on a sample size that participated in each IFI version in our discussion section. See Line 351-352. |
| 11. Line 207, “RA”: Some of these acronyms in the methods are a bit confusing, they mostly refer to the authors but now also to staff. In general, if I do not use a term that often, then I prefer to use the whole term instead of the acronym. I would consider this for "research assistant" in this manuscript. | Please see a revision Line 221-223. |
| 12. Line 210, revise “handed in” to “handed out.” | Please see a revision Line 223. |
| 13. Line 231, “Inclusivity in global research”: why is this mentioned here? Is this a requirement of the journal? If not, consider omitting. | Yes, it is required by the journal.  We have indicated “Inclusivity in global research” in our manuscript text and Supplement 2. |
| DISCUSSION 14. Line 311, The study’s findings…”: Be careful of overstating the findings. Consider, "The study finds that the T-IFI has appropriate internal and external content validity. This study suggests that this is an appropriate linguistic and cross-cultural adaptation of the IFI survey." | Please see a revision Line 316-318. |
| 15. Line 240, “more likely to access more sources” please revise. | Please see a revision Line 358. |
| 16. Line 342, “In antenatal care services…”: This sentence is a little bit unclear. What is the utility of this scale in a clinical setting? To see who is interested in breastfeeding and tailoring education to them? To get pregnant women interested in breastfeeding? Can this scale aid in getting Thai women to breastfeed and helping Thai women overcome barriers to breastfeeding exclusively for the first 6 months of life? | Please see a revision Line 362-364. |
| 17. Good job mentioning the need for caution and more reliability/validity testing in lines 347-350! | Thank you. |
| 18. Line 353, “exclusive breastfeeding,” add “among Thai women.” | Please see a revision Line 374. |

We appreciate your thoughtful comments and suggestions. We believe through the revision process; the manuscript has been improved. Thank you in advance for your consideration.

We look forward to hearing from you.

Sincerely,

Authors
